# In-silico analysis and transformation of OsMYB48 transcription factor driven by CaMV35S promoter in model plant – *Nicotiana tabacum* L. conferring abiotic stress tolerance

**DOI:** 10.1080/21645698.2024.2334476

**Published:** 2024-03-29

**Authors:** Yumna Ahmad, Saqlain Haider, Javed Iqbal, Sana Naseer, Kotb A. Attia, Arif Ahmed Mohammed, Sajid Fiaz, Tariq Mahmood

**Affiliations:** a Department of Plant Sciences, Faculty of Biological Sciences, Quaid-i-Azam University Islamabad, Islamabad, Pakistan; b Plant and AgriBiosciences Research Centre, Ryan Institute, University of Galway, Galway, Ireland; c Department of Botany, Bacha Khan University, Charsadda, Pakistan; d Department of Biochemistry, College of Science, King Saud University, Riyadh, Saudi Arabia; e Department of Plant Breeding and Genetics, The University of Haripur, Haripur, Pakistan

**Keywords:** Abiotic stress tolerance, crop improvement, crop yield, MYB transcription factor

## Abstract

Global crop yield has been affected by a number of abiotic stresses. Heat, salinity, and drought stress are at the top of the list as serious environmental growth-limiting factors. To enhance crop productivity, molecular approaches have been used to determine the key regulators affecting stress-related phenomena. MYB transcription factors (TF) have been reported as one of the promising defensive proteins against the unfavorable conditions that plants must face. Different roles of MYB TFs have been suggested such as regulation of cellular growth and differentiation, hormonal signaling, mediating abiotic stress responses, etc. To gain significant insights, a comprehensive in-silico analysis of OsMYB TF was carried out in comparison with 21 dicot MYB TFs and 10 monocot MYB TFs. Their chromosomal location, gene structure, protein domain, and motifs were analyzed. The phylogenetic relationship was also studied, which resulted in the classification of proteins into four basic groups: groups A, B, C, and D. The protein motif analysis identified several conserved sequences responsible for cellular activities. The gene structure analysis suggested that proteins that were present in the same class, showed similar intron-exon structures. Promoter analysis revealed major cis-acting elements that were found to be responsible for hormonal signaling and initiating a response to abiotic stress and light-induced mechanisms. The transformation of OsMYB TF into tobacco was carried out using the *Agrobacterium*-mediated transformation method, to further analyze the expression level of a gene in different plant parts, under stress conditions. To summarize, the current studies shed light on the evolution and role of OsMYB TF in plants. Future investigations should focus on elucidating the functional roles of MYB transcription factors in abiotic stress tolerance through targeted genetic modification and CRISPR/Cas9-mediated genome editing. The application of omics approaches and systems biology will be indispensable in delineating the regulatory networks orchestrated by MYB TFs, facilitating the development of crop genotypes with enhanced resilience to environmental stressors. Rigorous field validation of these genetically engineered or edited crops is imperative to ascertain their utility in promoting sustainable agricultural practices.

## 1. Introduction

Regulation of gene expression is fundamental to biological phenomena such as development, differentiation, growth, and stress responses. Transcriptional regulation of genes is a widespread phenomenon among living organisms and is dependent on the binding of transcription factors (TF), a group of proteins acting as a cis-element at the promoter region of target genes and enhancer regions of nearby genes.^1^ This binding then paves the way for the recruitment of other elements of transcription such as basal transcription factors and RNA polymerase. From the outlook of protein structure, transcriptional factors are made up of four parts: a transcriptional regulatory domain (TRD), a DNA-binding domain (DBD), a nuclear localization signal (NLS), and an oligomerization site.^2^ The characteristics and structure of transcriptional factors depend upon these functional regions.^3^ Plants respond to stress events by initiating stress-responsive gene expression which is regulated by a network of transcription factor families. In recent times several stress-induced transcription factors have been identified that participate in signal transduction pathways activating a whole array of genes under their control leading to plant adaptation to stress conditions.^4^

MYB transcription factors are a diverse group of regulatory proteins that are found in eukaryotes. They constitute the huge family of transcriptional factors in plants and interact with other transcription factors as well. The basic role of MYB proteins is in the regulation of plant primary and secondary metabolism, cell growth and differentiation, nutrient deficiency signaling, trichome development, and environmental stress responses.^5,6^ These proteins are found abundantly in plant species, and the function of multiple MYB proteins has been thoroughly investigated utilizing genetic and molecular techniques. These regulatory proteins provide an excellent opportunity to enhance plant stress tolerance due to their potential to modify the expression of several downstream genes.^7^

Advances in biotechnology have enabled the study of genes or the manipulation of desired pathways through genetic transformation. Transformation methods, including Agrobacterium-mediated transformation, biolistics, and CRISPR/Cas9 genome editing, enable precise genetic modifications to enhance stress tolerance. Concurrently, omics approaches such as genomics, transcriptomics, proteomics, metabolomics, and phenomics provide deep insights into the genetic, protein, metabolite, and phenotypic layers of stress response. These technologies collectively facilitate the identification and manipulation of key genes and pathways, leading to the development of crop varieties better equipped to withstand environmental stresses.

So far, research into the function of MYB proteins has been mostly carried out for the development of transgenic plants with increased resistance to drought, salinity, chilling, and heavy metal stress.^8^ However recent studies by^9–11^ presented that MYB proteins participate in heat stress-responsive gene expression leading to plant tolerance to high temperatures. Hence these transcription factors are excellent candidates for the development of thermotolerant crop plants.

However, an extensive literature review predicted that the role of OsMYB in a transgenic dicot plant and comparative analysis of OsMYB48 between monocots and dicots has not been studied at large, despite its significance in biological roles. Hence, the present study aims to carry out the transformation of the OsMYB gene in the wild tobacco plant and its genome-wide comparison using various in-silico approaches.

## 2. Materials and Methods

### 2.1. Computational Analysis

#### 2.1.1. Sequence Retrieval

The genomic sequence and coding sequence (CDS) of 21 dicots and 10 selected monocot plants were retrieved from NCBI (https://www.ncbi.nlm.nih.gov/). The essential data such as the gene’s location on the chromosome and the strand position of the gene was retrieved from Ensembl Plants (https://plants.ensembl.org/index.html) database. The corresponding protein sequences were obtained from the UniProt database. The proteins were subjected to conserved domain search (CD-search) in NCBI, to check the similarity index of domains and superfamily.

#### 2.1.2. Protein Domain Analysis

The MYB-DNA binding domains were identified by protein sequences that were subjected to analysis using an online tool SMART (http://smart.embl-heidelberg.de/) and were commanded to search for PFAM domains in the sequences. The results were then beautified using GeneDoc software.

#### 2.1.3. Phylogenetic Analysis

A phylogenetic tree was constructed to analyze the phylogenetic bond between OsMYB with corresponding genes. Protein sequences were aligned using Clustal W with default parameters. The tree was constructed via Neighbor-joining (NJ) method, using the Poisson model and pairwise deletion, with a bootstrap value of 1000 with the Molecular Evolution Genetic Analysis 11 (MEGA 11) tool.^12^

#### 2.1.4. Multiple Sequence Alignment

To find the sequence similarity, proteins of all MYB genes with default parameters were aligned using ClustalW (https://www.genome.jp/tools-bin/clustalw/). The aligned proteins were then edited and colored in GeneDoc software based on their conserved regions.

#### 2.1.5. Protein Motif Analysis

The conserved protein motifs responsible for different functions were identified using the Multiple Em for Motif Elicitation (MEME) server using default parameters.^13^ The threshold value for finding motifs was set to 30 conserved motifs.

#### 2.1.6. Promoter Analysis

The promoter sequence upstream 3000 bp of each gene was retrieved from NCBI and was subjected to evaluate the cis-regulatory elements by PlantCARE.^14^

#### 2.1.7. Gene Structure Analysis

The intron/exon sites of HSFs were investigated by aligning the CDS to its corresponding genomic DNA sequence using Gene Structure Display Server v2.0 (GSDS).^15^ The Newick tree generated from the phylogenetic analysis was exported into the GSDS input page to organize genes according to their phylogenetic relationships. Furthermore, the introns were represented by a black line, exons with red boxes while upstream/downstream regions with gray boxes.

#### 2.1.8. Physiochemical Properties

The physiochemical properties of proteins such as amino-acid length, molecular weight (Mw), and isoelectric point (pI), were determined using the ProtPram server. All the default parameters were set to mean values.^16^

#### 2.1.9. Subcellular Localization

The subcellular localization of targeted genes was evaluated through CELLO server v2.5 and UniProt.^17^

### 2.2. Transformation

The vector harboring the OsMYB48 gene was already present in the laboratory and was used for transformation purposes. The T/A cloning vector Pcambia3300 contained the gene of interest along with Kanamycin as a selection marker.

#### 2.2.1. Preparation of Electro-Competent Cells of DH5α

The strain of *E. coli*, DH5α was streaked on solid Luria Broth (LB) media using its glycerol stock. Streaked petri plates were incubated at 37°C, overnight. After obtaining discrete colonies, a single colony of DH5α was inoculated in 5 ml of LB broth and was kept at 37°C in an incubator shaker, overnight. To achieve the OD600 at 0.5–0.8, 45 ml LB broth was added to the starter culture and placed again in the incubator shaker at 37°C. The OD600 was checked with a spectrophotometer and the culture was further processed at an OD600 of 0.5–0.8. In 50 ml falcon tubes, the grown bacterial cells were pelleted out by centrifuging at 3000 rpm, at 4°C, for 15 minutes. After centrifugation, the supernatant was discarded, and the pellet was cleaned with 15% ice-cold glycerol and again centrifuged at 3000 rpm for 15 minutes. This washing technique was repeated again and again (at least three times) for the proper removal of excess glycerol and the bacterial pellet was re-suspended in 5 mL of 15% glycerol, and 50 µl aliquots were produced and kept at −80°C for later use. To test cell viability, 10 µl and 50 µl of competent cells were dispersed on the solid LB media plates after a 16-hour incubation at 37°C.

#### 2.2.2. Electroporation of Transformed Plasmid into DH5α Competent Cells

For electroporation, 50 µl of electrocompetent cells (DH5α) were thawed on ice and shifted to a cold cuvette along with 2 µl of transformed DNA. The solution was mixed gently and kept on ice for approximately 10 minutes. In Porator, electroporation is carried out at a specified voltage (2.5 kV/0.2 cm). These electroporated cells were resuspended immediately in 1 ml of liquid LB media in Eppendorf tubes (autoclaved) and were incubated at 37°C for 1 hour at 200 rpm with continuous shaking. The transformed mixture was spread on LB agar media containing selection marker kanamycin (50 mg/L) and incubated at 37°C for 12 hours. Non-transformed electrocompetent cells of DH5α were also streaked on LB agar media with kanamycin, as a negative control.

#### 2.2.3. Plasmid Isolation and PCR Confirmation

Plasmid from *E. coli* (DH5α) was extracted via protocols established by Sambrook and Russell, (2001)^18^ with few modifications. After 16–20 hours, single colonies were hand-picked and inoculated in 2 ml liquid LB broth containing kanamycin (50 mg/L). The inoculated broth was kept for overnight incubation at 250 rpm, at 37°C with constant shaking. The culture was then transferred into 1.5 ml autoclaved Eppendorf tubes and was centrifuged at 12,000 rpm, at 4°C for 5 minutes. The pellet obtained was resuspended after discarding the supernatant by adding 150 µl of ice-cold Solution-I (Resuspension buffer) and 50 µl RNAase A and the solution was vortexed.

Solution-II (Lysis solution) (200 µl) was added to the solution, inverted 5–10 times for proper mixing, and placed on ice for a maximum of 10 minutes. Then 300 µl of Solution-III (Neutralization buffer) was added. The Eppendorf was then vortexed for 10 seconds to disperse the solution III through bacterial viscous lysate and stored the Eppendorf on ice for 5 minutes again.

The contents were centrifuged at 14,000 rpm at 4°C for 5 minutes. The supernatant was separated and shifted to a new Eppendorf tube and the pellet was discarded. The supernatant was then vortexed by adding an equal volume of Phenol: Chloroform (24:1) and centrifuged again for 5 minutes at 14,000 rpm. A clear supernatant was observed and carefully transferred to the fresh Eppendorf tube. By adding an equal percentage of isopropanol, the double-stranded DNA was precipitated. After keeping Eppendorf tubes at room temperature for 10 minutes, the mixture was centrifuged at the speed of 14,000 rpm for 5 minutes with a set temperature of 4°C. The supernatant was carefully decanted, and the DNA was washed with 70% ethanol that settled at the bottom in the form of a pellet. The pellet dried by keeping Eppendorf tubes inverted in the air on sterilized filter paper and was suspended in 100 µl TE buffer and stored at −20°C immediately for further use. The isolated plasmid from transformed DH5α was confirmed by running a polymerase chain reaction (PCR) with OsMYB48-specific primers.

#### 2.2.4. *Preparation of* Agrobacterium *(LBA4404) Electro-Competent Cells*

The electrocompetent cells of *Agrobacterium* strain LBA4404 were prepared with the same methodology as used in the preparation of DH5 cells, except that the incubation temperature was 28°C and the timeframe was 24–48 hours, respectively. The competent cells were spread on LB media plates and were placed under incubation at 28°C for 24–48 hours, to check their viability.

#### 2.2.5. Agrobacterium Transformation with Recombinant Vector

Electrocompetent cells of *Agrobacterium* (LBA4404) cells were electroporated with recombinant vector through electroporation, using the same methodology as used DH5α cells. For screening, a 50–100 µl transformed mixture was spread on LB media plates containing 50 mg/L of kanamycin and incubated for 48 hours at 28°C. Colony PCR was used to confirm the transformed colonies.

#### 2.2.6. Agrobacterium-Mediated Transformation of Nicotiana tabacum

The protocol suggested by Horsch et al.,^19^ was followed to transfer the tobacco plants with OsMYB48 using *Agrobacterium*, with few modifications.

#### 2.2.7. Selection of Plant Material

To perform *Agrobacterium*-mediated transformation, the tobacco species, *Nicotiana tabacum* var. Virginia was selected as experimental material. Seeds of tobacco were kindly provided by the National Agriculture Research Centre (NARC), Islamabad, Pakistan.

#### 2.2.8. Seed Sterilization

Seeds of tobacco plants were surface sterilized by repeated washing with double distilled water and 70% ethanol, followed by one-time washing with 30% hypochlorite solution. Lastly, the seeds were washed with double distilled water and placed on filter paper to dry.

#### 2.2.9. Seed Germination and Plant Growth

For germination, seeds were dried and sterilized on filter paper (autoclaved) and placed on plates containing only MS media. Plates were placed then in a growth chamber under optimal conditions of 27°C temperature and a 16:8 light/dark cycle. Young seedlings were observed after two weeks. Plants when reached the 2–3 leaf stage, were shifted to culture bottles with simple MS media for shoot elongation and rooting.

#### 2.2.10. Explant Preparation for Transformation

The leaves of a 1-month-old tobacco plant were cut into small leaf discs without the midrib ranging from 1–2 cm and were placed adaxially on solidified full-strength MS media for pre-culture. Nodal explants were also cut into small pieces and placed alongside leaf explants on simple MS media. Approximately 10–15 explants were sited on a single MS media, sealed the plates with parafilm, and incubated at a 16/8-hour photoperiod for 48 hours.

#### 2.2.11. Infection and Co-Cultivation

Recombinant *Agrobacterium*’s strain LBA4404 was streaked on a solid LB media petri plate from its glycerol stock and was kept in the dark at 28°C for 24–48 hours. After obtaining colonies, a single colony was taken and grown in 25 ml liquid LB media containing 50 mg/L Kanamycin and acetosyringone (40 g/L) to increase the bacterial virulence by incubating at 28°C with constant shaking at 250rpm for 36–48 hours. When the OD_600_ of suspension reached between 0.5–0.8, the suspension was transferred to the 50 ml falcon tubes and was centrifuged at 3000 rpm for 10 minutes. The bacterial pellet was resuspended in the hormone-free MS liquid media (35 ml) and the supernatant was discarded carefully. Leaf explants were placed inside the falcon tube and the tube was swirled gently 2–3 times for better attachment of bacteria to explants. Explants were kept suspended in the solution for 10 minutes and then removed by using forceps and placed on hormone-free MS media petri plates. The petri dishes were sealed with parafilm, covered with aluminum foil, and kept in a growth chamber at 25°C for co-cultivation by providing a 16-hour light and 8-hour darkness cycle for two days.

#### 2.2.12. Selection of Transformants

Following co-culture, the infected explants were transferred to the selection media i.e., MS-Hormonal (MSH) media containing cefotaxime 500 mg/L and kanamycin 50 mg/L. The media’s pH was set to 5.8 as per the growth requirement. After autoclaving, the selection media, cefotaxime, and kanamycin were added. Cefotaxime was utilized to prevent *Agrobacterium*’s overgrowth, while kanamycin was applied to select the transformants. The selected explant transformants were subcultured on the fresh selection media, every 14 days.

#### 2.2.13. Regeneration and Shooting

After 20 to 25 days, the putatively transformed explants developed calli at the cut edges of the explants. After about 3–4 weeks, adventitious shoots began to appear. Regenerated shoots were afterward transferred to large jars for appropriate growth, using the same selection media and growing conditions as before.

#### 2.2.14. Rooting of Transformed Plants

The shoots were excised from the explant when they developed two to three internodes and transferred to large ampules containing hormone-free solid MS medium for optimal rooting.

#### 2.2.15. Confirmation of Transgenic Plants

First, transgenic plants were selected on selection media that contained Kanamycin (50 mg/L) and growth hormones, and plants that remained green and showed putative growth were later confirmed by PCR.

### 2.3. Molecular Analysis

#### 2.3.1. Genomic DNA Isolation

For the confirmation of transgenic plants, both wild-type and transgenic plants’ total genomic DNA was isolated following the CTAB (Cetyl Trimethyl Ammonium Bromide) method proposed by Murray and Thompson.^20^ Leaves were ground in a pestle and mortar by adding liquid nitrogen into fine powder. The powder was shifted to a 1.5 ml Eppendorf tube along with 500–700 µl of pre-warmed CTAB buffer. Samples were vortexed for proper mixing and were placed in a water bath for an hour. After that, an equal amount of phenol: chloroform: isoamyl alcohol (25:24:1) was added to the tube and centrifuged the solution at 14,000 rpm for 15 minutes. The supernatant was shifted to another sterile Eppendorf tube and discard the pellet. An equal amount of pre-chilled isopropanol was mixed in a supernatant to precipitate the genomic DNA and the tube was incubated overnight on ice. The contents were centrifuged at 14,000 rpm for 15 minutes to collect the pellet and discard the supernatant. The pellet obtained was washed with 70% ethanol, resuspended in the 30–50 µl TE buffer, and stored at −20°C for future use. To confirm the presence of DNA, the gel electrophoresis method was used.

#### 2.3.2. PCR Confirmation

The isolated genomic DNA was subjected to PCR for the confirmation of transformation. The 25 µl of PCR mixture was prepared. Amplification was processed using a Gradient Multigene Thermal Cycler (Labnet). The amplification reaction had 35 cycles of denaturation at 95°C for 5 minutes, 54°C for 90 seconds, 72°C for 60 seconds, and 72°C for 20 minutes.

The primer sequences used for the amplification are given below.

OsMYB48 (Forward): 5ʹ ATCATGAGGACCAGCACCAA 3ʹ

OsMYB48 (Reverse): 5ʹ AGGTAGCGAATAATCCGAGCA 3ʹ

#### 2.3.3. Gel Electrophoresis and Imaging

To visualize the results of PCR, run the PCR product on 1% agarose gel along with 1kb ladder DNA for the confirmation of the size of the band. It was later visualized in the Gel Documentation System under UV light.

## 3. Results

### 3.1. Computational Analysis

#### 3.1.1. Identification of MYB Genes

**Figure 1.**
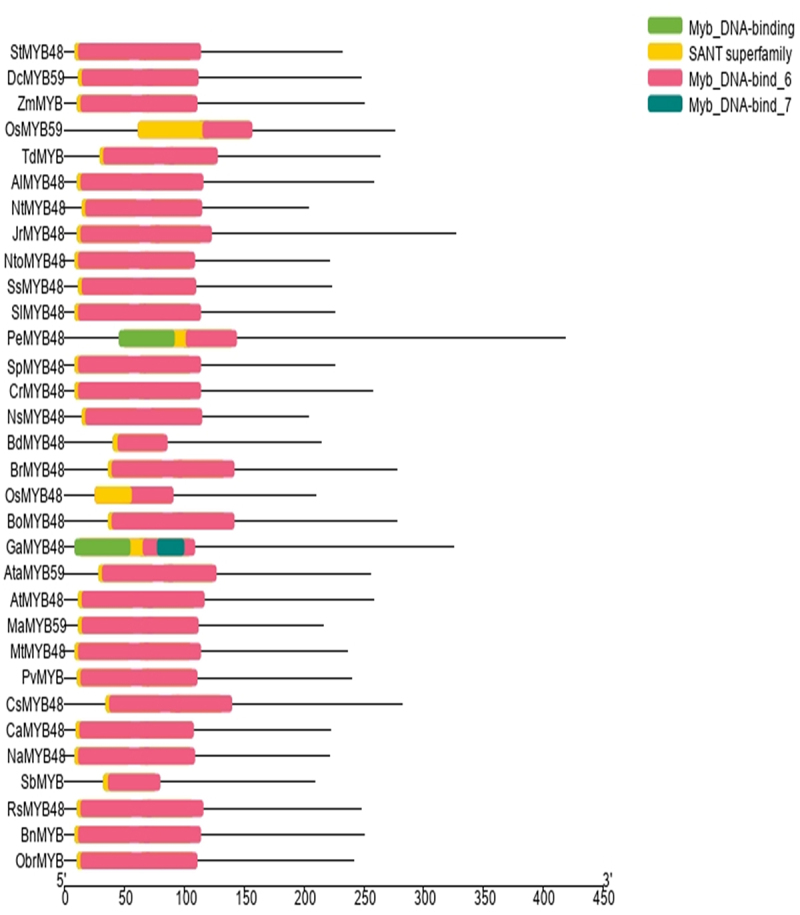
Conserved domains present in OsMYB48 and relative MYB proteins.

In the present study, the gene and protein sequence of Oryza sativa japonica group’s OsMYB48 was blasted against multiple dicot and monocot plants in NCBI. Genomic sequence, coding sequence (CDS), and promoter sequence of genes showing the highest similarity to the sequence were retrieved from NCBI and Ensembl Plants. The protein sequences were obtained from the UniProt. The protein sequences obtained were subjected to a Conserved Domain Database (CDD) search in NCBI to find the conserved protein domains specific to the MYB transcription factor family. Each protein confirmed the presence of highly conserved MYB-DNA binding domains. Subsequently, 1 sequence each of 21 dicots (*Arabidopsis thaliana, Arabidopsis lyrata, Brassica rapa, Brassica oleracea, Brassica napus, Camelina sativa, Capsicum annuum, Capsella rubella, Gossypium arboretum, Juglans regia, Medicago truncatula, Nicotiana sylvestris, Nicotiana attenuate, Nicotiana tomentosiformis, Nicotiana tabacum, Phalaenopsis equestris, Raphanus sativus, Salvia splendens, Solanum pennellii, Solanum lycopersicum*, and *Solanum tuberosum*) and 10 monocots (*Aegilops tauschii, Brachypodium distachyon, Dioscorea cayenensis, Musa acuminata, Oryza sativa japonica, Oryza brachyantha, Panicum virgatum, Sorghum bicolor, Triticum dicoccoides*, and *Zea mays*) plant species were selected for analysis. The gene accession numbers of MYB genes along with their plant species have been presented in Table 1. The conserved domains of these proteins i.e., the SANT domain and MYB-DNA binding domains are presented in Figure 1.

**Table 1.** The *MYB* genes with their identification numbers and respective plant species.

Gene	Gene ID/Accession number	Plant species
*OsMYB48*	Os01g0975300/BAD87845	*Oryza sativa japonica group*
*AlMYB48*	XP_002875766	*Arabidopsis lyrata*
*AtMYB48*	NP_190199	*Arabidopsis thaliana*
*BnMYB*	NP_001303156	*Brassica napus*
*BoMYB48*	XP_013625304	*Brassica oleracea*
*BrMYB48*	XP_009149988	*Brassica rapa*
*CsMYB48*	XP_010503226	*Camelina sativa*
*CrMYB48*	XP_006291710	*Capsella rubella*
*CaMYB48*	XP_016548288	*Capsicum annuum*
*GaMYB48*	XP_017613576	*Gossypium arboreum*
*JrMYB48*	XP_018806931	*Juglans regia*
*MtMYB48*	XP_003597326	*Medicago truncatula*
*NaMYB48*	XP_019263011	*Nicotiana attenuata*
*NsMYB48*	XP_009804412	*Nicotiana sylvestris*
*NtMYB48*	XP_016495219	*Nicotiana tabacum*
*NtoMYB48*	XP_009590049	*Nicotiana tomentosiformis*
*PeMYB48*	XP_020592623	*Phalaenopsis euqestris*
*RsMYB48*	XP_018436504	*Raphanus sativus*
*SsMYB48*	XP_041991308	*Salvia splendens*
*SlMYB48*	NP_001310217	*Solanum lycopersicum*
*SpMYB48*	XP_015077480	*Solanum pennellii*
*StMYB48*	XP_015165693	*Solanum tuberosum*
*AtaMYB59*	XP_045089944	*Aegilops tauschii subsp. strangulata*
*BdMYB48*	XP_024314946	*Brachypodium distachyon*
*DcMYB59*	XP_039144280	*Dioscorea cayenensis subsp. rotundata*
*MaMYB59*	XP_009404618	*Musa acuminata subsp. malaccensis*
*OsMYB59*	XP_015621159	*Oryza sativa japonica*
*ObrMYB*	XP006664115	*Oryza brachyantha*
*PvMYB*	XP_039839852	*Panicum virgatum*
*SbMYB*	XP_021321599	*Sorghum bicolor*
*TdMYB*	XP_037416338	*Triticum dicoccoides*
*ZmMYB*	NP_001140590	*Zea mays*

#### 3.1.2. Multiple Sequence Alignment

**Figure 2.**
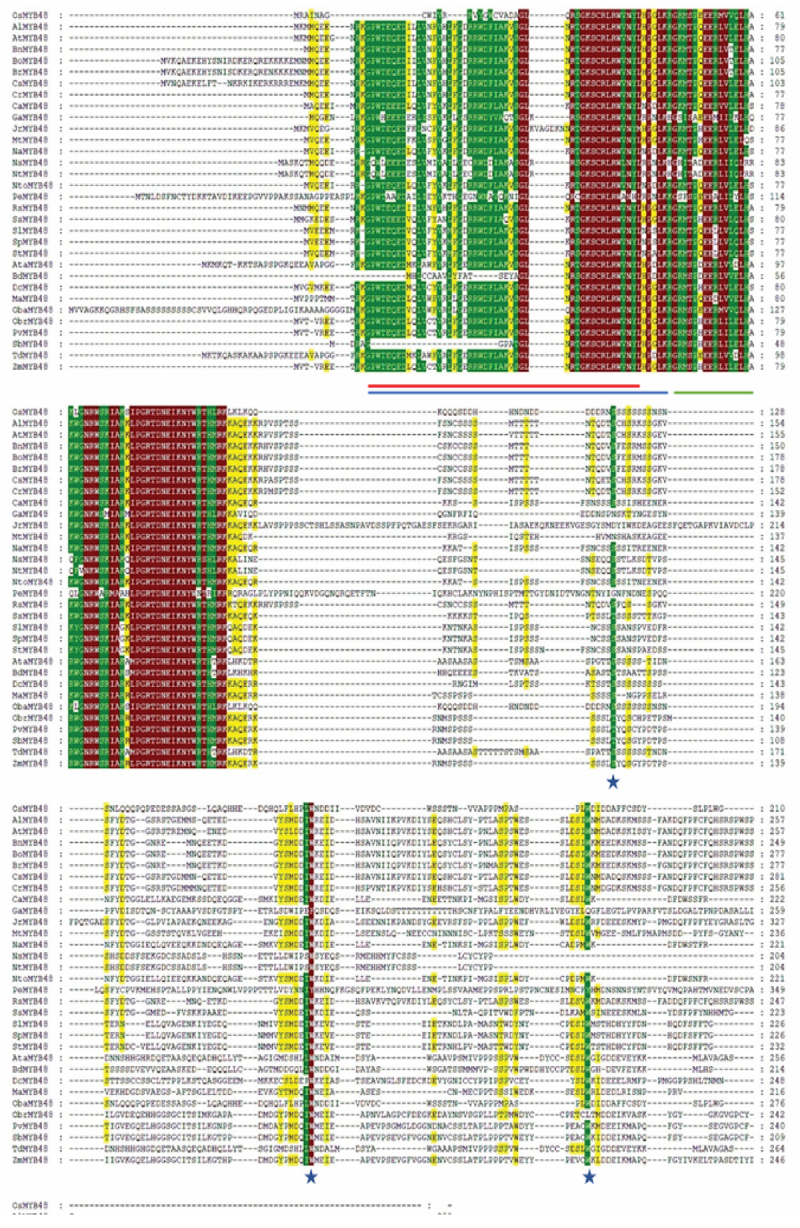
Multiple sequence alignment of OsMYB48 with MYB proteins of monocots and dicots. The red line represents the conserved SANT domain. The blue line represents the repeats of the conserved domain of R2-MYB, and the green line represents the repeats of the R3-MYB conserved domain. Stars indicate the presence of conserved amino acid residues in the proteins.

To identify the conserved domain sequence within each repeat of the OsMYB48 along with other MYB proteins, multiple sequence alignment by Clustal W was performed. In the present study, the analysis performed by Du et al.^21^ helped to identify the highly conserved MYB and SANT domains in the protein sequences (Figure 2). The SANT domain was found to be located near the N-terminal of MYB proteins and consists of approximately 50 amino acids. The basic regions of MYB DNA-binding domains had approximately 105 amino-acid residues, with rare deletions and insertions. R2-R3 MYB DNA-binding domains overlapped and followed the SANT domain. Both R2 and R3-MYB domain sequences indicated the existence of three helixes of the HTH domain, which is known for highly specific binding.

Consistent with earlier reports, which reported the presence of characteristic amino-acids in the R2-R3 MYB repeats of Arabidopsis, OsMYB and its homologs were also found to contain such as tryptophan (Trp) residues, which are a key player in sequence-specific DNA binding.^22^ Other than that, highly conserved amino-acid residues were found in addition to the Trp residues (W). These residues included Glycine (G), Proline (P), Threonine (T), Glutamic acid (E), Glutamine (Q), Leucine (L), Valine (V), Arginine (R) Aspartic acid (D), Phenylalanine (F), Isoleucine (I), Lysine (K) and Serine (S) in R2-MYB repeats. Glycine, lysine, Serine, Leucine, Arginine, Proline, Methionine (M), Glutamic acid, Histidine (H), Tryptophan, Tyrosine (Y), and Asparagine (N) were observed to be highly conserved in R3-MYB repeats. In each repeat, between the 2nd and 3rd conserved Trp residues, the alignment predicted the distribution of significant conserved MYB domain residues. As a result, the MYB domain in the first half of each repeat was discovered to be less conserved, while the third helix of the HTH domain was revealed to be highly conserved across all proteins in each repeat. Overall, there seems to be a significant similarity between OsMYB and other MYB proteins.

#### 3.1.3. Protein Motif Analysis

**Figure 3.**
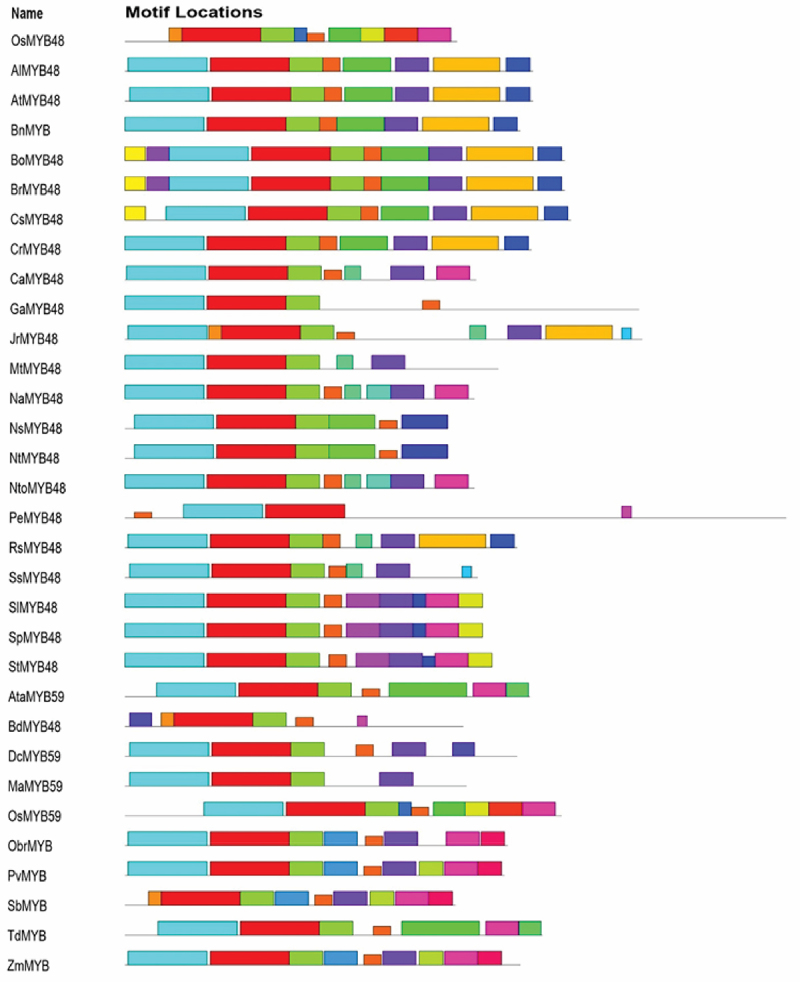
Identification of conserved motifs through MEME. Each motif is represented by a unique color.

**Figure 4.**
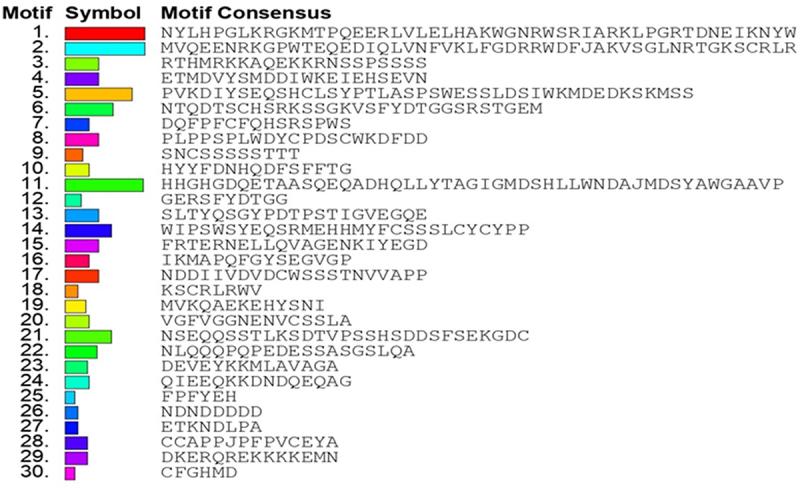
Conserved motifs sequence as identified by MEME.

To identify the conserved motifs/regions in OsMYB48 protein with comparison to other MYB proteins, MEME was employed. In total, 30 motifs were identified in both dicot and monocot plants (Figure 3). The sequence of these motifs has been presented in Figure 4. Motifs 1 and 2 have the most amino acid residues (50), followed by motif 11 (49), and motif 5 (42). Motifs 1 and 3 represented the R3-MYB domain and were found to be present in all proteins. Motif 2 represents the R2-MYB domain and was found in all proteins except OsMYB48, SbMYB, and BdMYB48. The sequence is similar to the first helix of R2 repeat, which has been found to be less conserved during multiple sequence alignment. Although motif 18 with 8 residues has been determined near the N-terminal of these proteins where lies the R2-MYB domain the sequence shows high similarity with the third helix of the R2-MYB domain. The presence of long-conserved residues in the aligned sequences can reveal the conserved topology of MYB proteins across species. Motif 4 with 21 residues was found in all proteins except OsMYB48, GaMYB48, NsMYB48, NtMYB48, AtaMYB59, BdMYB48, OsMYB59, and TdMYB. Motif 5 with 42 residues was only predicted in 9 sequences, all dicot plants. This region could be a consensus sequence in dicotyledonous MYB proteins. Other motifs were found in variations in all protein sequences and showed less similarity. OsMYB48 and OsMYB59 showed high similarity and shared similar motifs except motif 2 in OsMYB59. A total of 9 motifs were found in the OsMYB48 protein sequence, of which most of the motif’s function is yet to be determined.

#### 3.1.4. Phylogenetic Analysis of MYB48 Proteins

**Figure 5.**
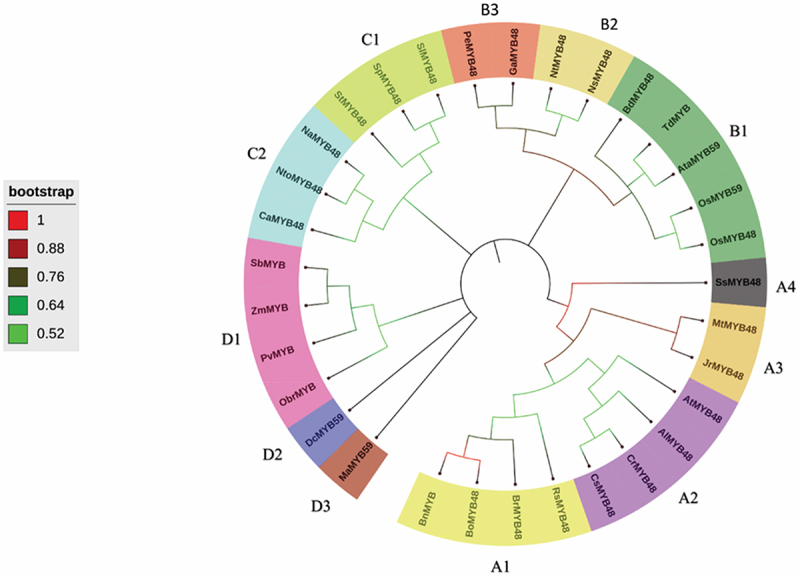
Phylogenetic tree of MYBs constructed by the neighbor-joining method.

A neighbor-joining phylogenetic tree incorporating both dicot and monocot plants was created to gain insight into the phylogeny and evolution of OsMYB48. MEGA 11 was used to create the phylogenetic tree, which was then refined with iTOL (Figure 5). The analysis performed by Islam et al.^23^ helped in tree annotation. Based on the tree topology, the created tree was divided into four basic groups: groups A, B, C, and D. Based on their evolutionary ties, these groups were further subdivided into sub-groups. In regard to gene structure and conserved functional domains, some genes from distinct groups may differ.

According to the phylogram, class A showed the maximum number of sub-classes, followed by class B, D, and C’s MYB proteins. The gene of interest OsMYB48 was classified into class B, based on its structural and physicochemical properties. The class A was divided into four sub-classes including A1 (BnMYB, BoMYB48, BrMYB48 and RsMYB48), A2 (CsMYB48, CrMYB48, AlMYB48, and AtMYB48), A3 (JrMYB48, and MtMYB48), and A4 (SsMYB48). The class B was divided into three sub-classes including B1 (OsMYB48, OsMYB59, AtaMYB59, TdMYB, and BdMYB48), B2 (NsMYB48, and NtMYB48), and B3 (GaMYB48, and PeMYB48). Class C was divided further into two sub-classes including C1 (SlMYB48, SpMYB48, and StMYB48), and C2 (NaMYB48, NtoMYB48, and CaMYB48). Class D was divided into three sub-classes including D1 (SbMYB, ZmMYB, PvMYB, and ObrMYB), D2 (DcMYB59), and D3 (MaMYB59).

According to the analysis, OsMYB48 grouped with other monocots, and dicot plants also grouped with other dicots. OsMYB48 showed a close relationship with OsMYB59 in the phylogram, depicting a close evolutionary relationship. However, it was observed that species from the same genus as *Nicotiana*, *Arabidopsis*, *Brassica*, and *Solanum* clustered into similar groups, which predicts the conservation of MYB proteins during evolutionary processes, both in monocots and dicots. OsMYB48’s clustering with other monocots suggests that it arises from the same lineage. However, the function of the gene cannot be predicted merely on tree topology. More molecular and genetic studies can explain the role of OsMYB48 during various conditions.

#### 3.1.5. Gene Structure Analysis

**Figure 6.**
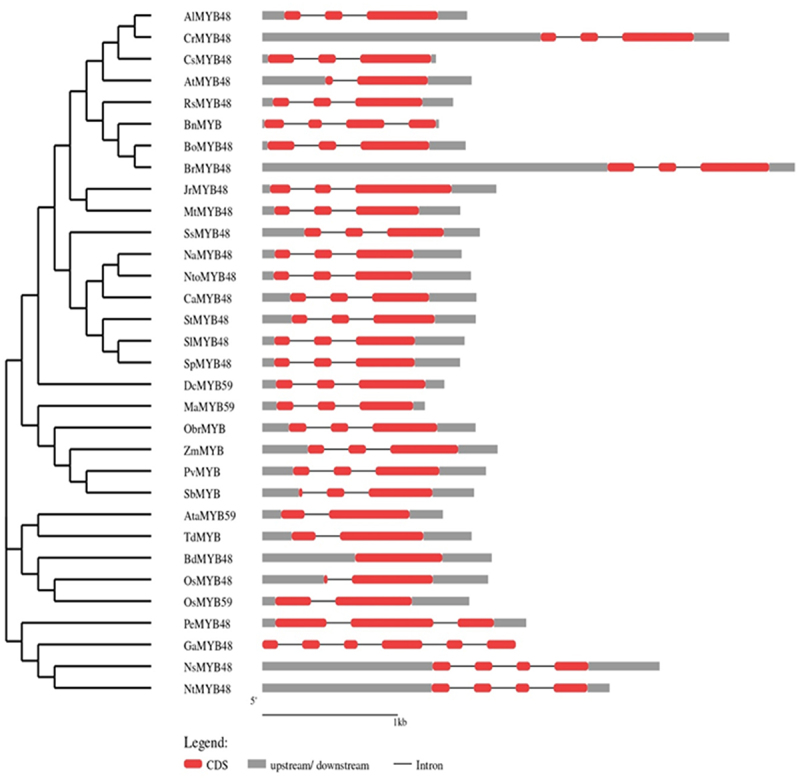
Gene structure of OsMYB48 along with various MYB genes of monocot and dicot plants. The intron is represented by the black line, coding sequence with a red box, while the untranslated region with a grey box. The length of the introns, exons, and untranslated sections is indicated by the scale bar at the bottom.

GSDS software was used to evaluate the intron-exon arrangement of all of the targeted MYB proteins to investigate the structural link between OsMYB48 and its related monocotyledonous and dicotyledonous MYB proteins. MYB genes from the same class and subclass have nearly equivalent intron-exon patterns with respect to intron count, intron phase, exon length, and overall gene length (Figure 6). The introns in class A ranged from 1 to 3. In OsMYB48, only one intron was found to be interrupting the coding sequence. Other members of sub-class B1 also showed one or no intron. The sub-class B2 members possessed two introns. While sub-class B3 showed a variation of introns, ranging from 2 to 5 introns. Both class C and D possessed two introns in each sequence. The GSDS analysis was in line with the phylogenetic relationships between OsMYB48 and other MYB proteins.

#### 3.1.6. Physio-Chemical Properties of OsMyb48 and Relative MYB Proteins

The physio-chemical properties of OsMYB48 and other targeted proteins such as chromosomal location, amino-acid length, iso-electric point (pI), and molecular weight (Mw), were investigated using Expasy, NCBI, and Ensembl Plants (Table 2). OsMYB48 was found to be present on the forward strand of chromosome number 1. It has 210 amino-acid residues. Its isoelectric point value was calculated to be 6.29 and Mw was determined as 24,075.62 g/mol. The chromosomal location of a few genes was not available yet.

**Table 2.** Physiochemical properties of selected *MYB* genes.

Gene	Chromosome	Locus position	Strand	Amino acid residues	Isoelectric point (pI)	Molecular weight (Mw)
*OsMYB48*	1	43096858–43097940	+	210	6.29	24,075.62
*AlMYB48*	N/A	12402012–12403343	+	258	9.31	29,864.63
*AtMYB48*	3	16945230–16946699	+	258	9.13	29,913.43
*BnMYB*	3	57119753–57120792	-	250	8.94	29,201.84
*BoMYB48*	3	47845466–47846476	+	278	9.44	32,731.84
*BrMYB48*	6	1122,896–11229824	-	278	8.99	32,666.69
*CsMYB48*	4	13309398–13310474	+	282	9.56	32,910.06
*CrMYB48*	N/A	6910203–6913437	+	257	9.15	29,742.31
*CaMYB48*	11	36160041–36161987	+	222	6.87	26,233.44
*GaMYB48*	12	34314300–34317621	+	325	7.70	36,930.67
*JrMYB48*	14	9845551–9847153	+	327	5.90	37,178.68
*MtMYB48*	2	47279597–47281017	-	236	6.20	27,425.67
*NaMYB48*	N/A	157990–159879	+	221	8.33	26,332.64
*NsMYB48*	N/A	11520–14582	+	204	6.66	23,640.32
*NtMYB48*	N/A	103232–105923	-	204	6.66	23,682.40
*NtoMYB48*	N/A	33912–35649	-	221	8.33	26,264.59
*PeMYB48*	N/A	106560–108317	+	418	6.64	47,258.97
*RsMYB48*	N/A	38570123–38571383	+	248	9.05	28,860.48
*SsMYB48*	7	8785890–8787322	-	223	9.35	26,093.51
*SlMYB48*	6	47160641–47162214	-	226	6.01	26,583.68
*SpMYB48*	6	57898287–57899827	-	226	6.01	26,502.58
*StMYB48*	N/A	81526–83287	-	232	6.10	27,068.19
*AtaMYB59*	3	624123398–624124705	-	256	8.91	28,581.01
*BdMYB48*	2	58910430–58912123	+	214	6.11	24,285.96
*DcMYB59*	18	22479435–22480665	+	248	8.89	28,328.17
*MaMYB59*	6	10243714–10244717	+	216	8.36	24,901.01
*OsMYB59*	1	43096382–43097941	+	276	6.86	31,029.37
*ObrMYB*	12	12318068–12319640	-	242	8.05	27,632.44
*PvMYB*	3	57078758–57080262	-	240	8.14	27,388.05
*SbMYB*	8	56140699–56142185	-	209	8.26	23,485.51
*TdMYB*	3	859468270–859469816	-	264	8.37	29,183.46
*ZmMYB*	3	128792069–128793670	+	250	8.54	28,674.60

#### 3.1.7. Cis-Acting Element Analysis

**Figure 7.**
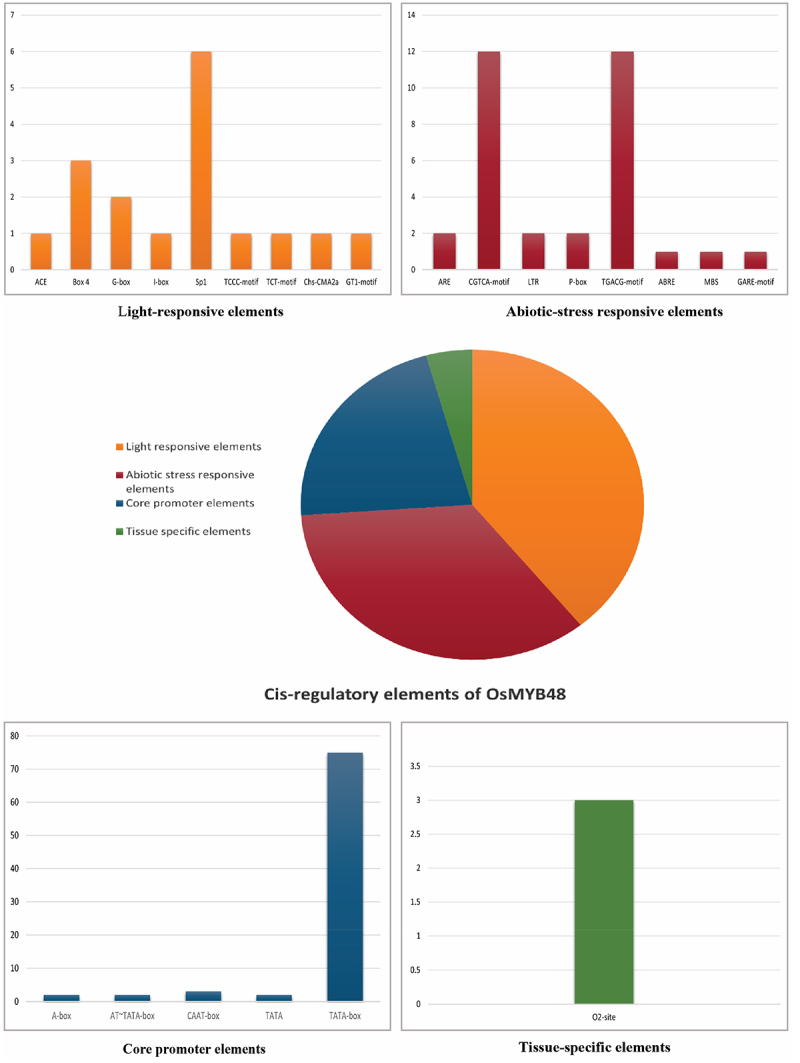
Cis-acting elements of OsMYB48 gene.

The cis-regulatory elements of OsMYB48 were identified by subjecting its promoter sequence (3000 bp upstream) to the promoter analysis in the PlantCARE database. Results predicted the presence of nine regulatory elements involved in light responsiveness; eight elements involved in abiotic stress responses; five core promoter elements; one tissue-specific element, and six elements conserved for MYB-binding and recognition activities (Table 3). Abiotic stress-responsive elements included the regulatory elements acting against hormonal responses of MeJA, Gibberellin, and abscisic acid and environmental stresses such as anaerobic induction, drought, and cold. The ratio of these elements in the promoter region has been presented in Figure 7. All of these elements suggest a potential role of OsMYB48 in stimulating abiotic stress responses and in cell growth and differentiation mechanisms.

**Table 3.** Cis-acting elements are found in the promoter region of *OsMYB48*..

Cis-acting element	Sequence	Copy number	Function
A-box	CCGTCC	3	Cis-acting regulatory element
ACE	GACACGTATG	1	Involved in light responsiveness
ARE	AAACCA	2	Required for the anaerobic induction
AT~TATA-box	TATATA, TATATAAA	9	Core promoter element
BOX 4	ATTAAT	3	Involved in light responsiveness
CAAT-box	CCAAT, CAAAT, CAAT	28	Common cis-acting element in promoter and enhancer regions
CCAAT-box	CAACGG	1	MYBHv1 binding site
CGTCA-motif	CGTCA	12	Involved in the MeJA-responsiveness
G-box	CACGAC	2	Involved in light responsiveness
I-box	gGATAAGGTG	1	Part of light responsive element
LTR	CCGAAA	2	Involved in low-temperature responsiveness
MYB	CAACAG	1	Core MYB-binding site
MYB recognition site	CCGTTG	1	-
MYC	CATGTG, CATTTG	2	-
MYB-binding site	CAACAG	1	-
MYc	TCTCTTA	1	-
O2-site	GATGACATGG	3	Involved in zein metabolism regulation
P-box	CCTTTG	1	Gibberellin-responsive element
Sp1	GGGCGGG	6	Light-responsive element
TATA-box	TATAAAAT	2	Core promoter element
TCCC-motif	TCTCCCT	1	Part of a light-responsive element
TCT-motif	TCTTAC	1	Part of a light-responsive element
TGACG-motif	TGACG	12	Involved in the MeJA-responsiveness
Chs-CMA2a	TCACTTGA	1	Part of a light responsive element
MBS	CAACTG	1	Involved in drought-inducibility
GT1-motif	GGTTAA	1	Light responsive element
GARE-motif	TCTGTTG	1	Gibberellin-responsive element
ABRE	ACGTG	1	Involved in the abscisic acid responsiveness

#### 3.1.8. Sub-Cellular Localization

The sub-cellular location of MYB proteins was identified and predicted by UniProt and CELLO server. The location of OsMYB48 and all other proteins was predicted to be in the nucleus of the cell. This suggests that these proteins play a significant role in core regulation and similar biological processes.

### 3.2. Functional Analysis

#### 3.2.1. Validation of Cloning of OsMyb48 by PCR

**Figure 8.**
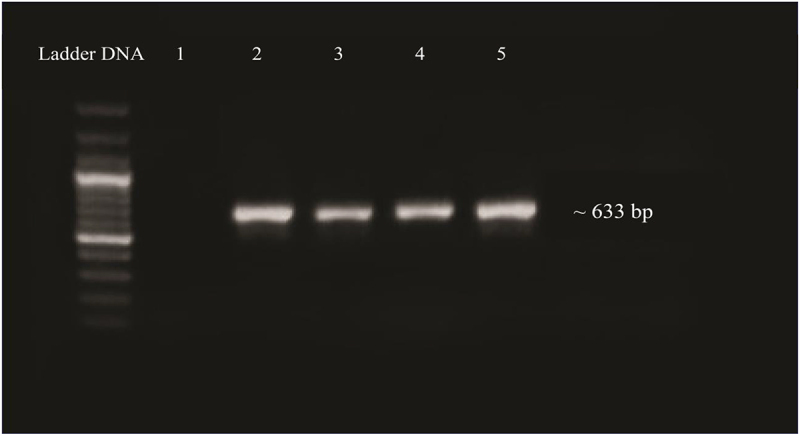
PCR confirmation of gene ligation in *E. coli* plasmid. Lane 1: negative control. Lane 2–5: amplified product of gene.

The T/A cloning vector containing the OsMYB gene was cloned into *E. coli* (DH5α) cells via electroporation method. The bacterial colonies obtained were cultured in LB media containing 50 mg/L kanamycin and incubated at 37°C, at 200 rpm shaking overnight to validate the cloned gene into *E. coli*. Isolated plasmid from bacterial culture was confirmed by PCR using gene-specific primers. On a 1% agarose gel, a product of 633 bp (Figure 8) was identified, confirming the cloning of the OsMYB48 gene.

#### 3.2.2. *Transformation of OsMyb48 into* Agrobacterium Tumefaciens

**Figure 9.**
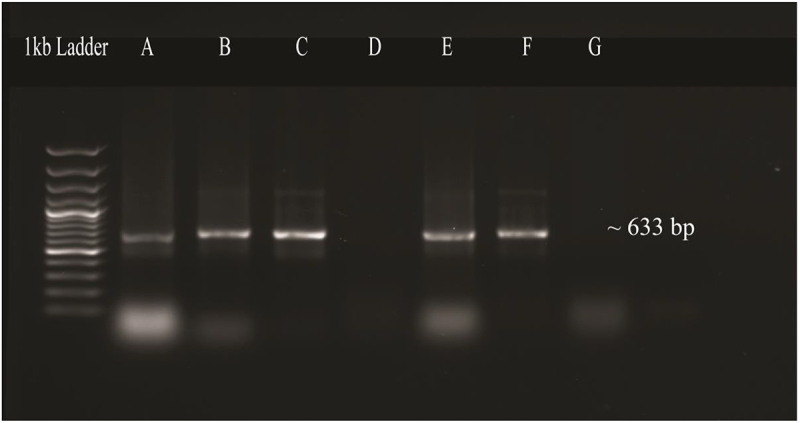
PCR confirmation of transformation of OsMYB48 into *agrobacterium tumefaciens*. Lane A, B, C, E: amplified products of *OsMYB48* gene. Lane D, G: negative control.

The OsMYB48 expression vector was transformed into the *Agrobacterium tumefaciens* strain (LBA4404) via the electroporation technique. The *Agrobacterium*-mediated transformation results were established by colony PCR by using the OsMYB48 gene-specific primers which resulted in the desired amplification (Figure 9).

#### 3.2.3. Agrobacterium*-Mediated Transformation of Tobacco with OsMyb48*

**Figure 10.**
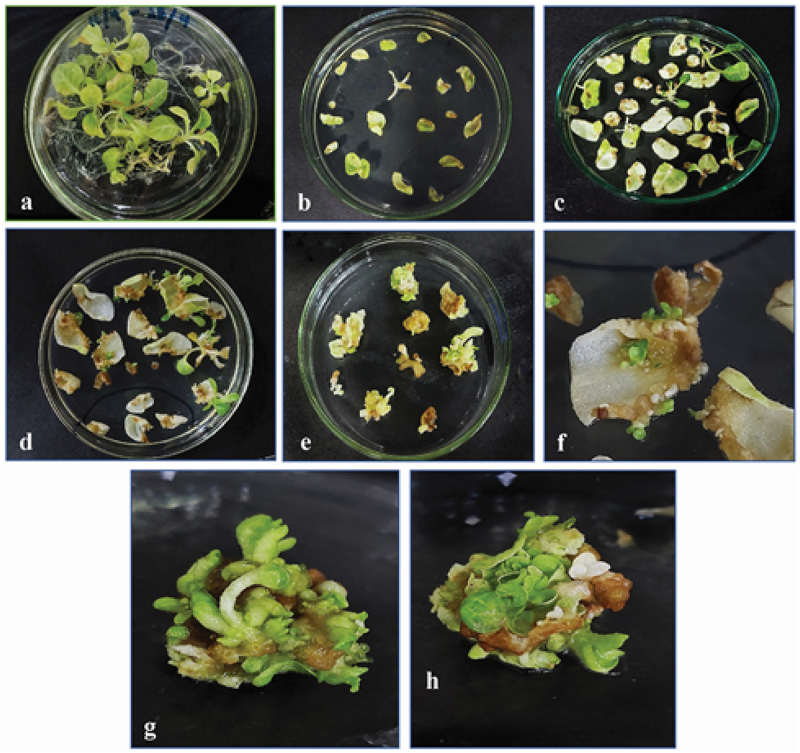
Regeneration of transformed tobacco from leaf and stem explants growing on selection media. (a) 2-month-old young tobacco plant, (b) explants placed on selection media after infection, (c, d) direct regeneration of new plants from stem explants, (e) calli emerging from cut edges of leaf explants, (f) small plants emerging from leaf explants, (g, h) 21 days old callus, showing shoot regeneration.

1-month-old tobacco plant’s young leaves were grown on MS media and were subjected to *Agrobacterium*-mediated transformation under controlled growth conditions and hormonal concentrations with selection marker Kanamycin (50 mg/L). After two weeks, the nodal explants and some leaf explants showed direct regeneration of the plant (Figure 10). Other explants started developing small bud-like calluses at their cut edges. Both regenerated plants and calluses were subjected to continuous shifting on fresh media every 2 weeks. In the form of small shootlets, the calli began to regenerate. After 4 weeks, the shootlets had grown to the point where they could be separated from callus and explant and shifted to large vessels for optimal growth. For rooting, regenerated shoots with at least two to three nodes were transferred to hormone-free MS media. After 15 days, rooting was observed. Later, these transgenic plants were shifted to the soil in small pots to obtain a mature plant under controlled conditions of 16:8 light/dark cycles at 25°C, in a greenhouse.

#### 3.2.4. Validation of Transgenic Plants

**Figure 11.**
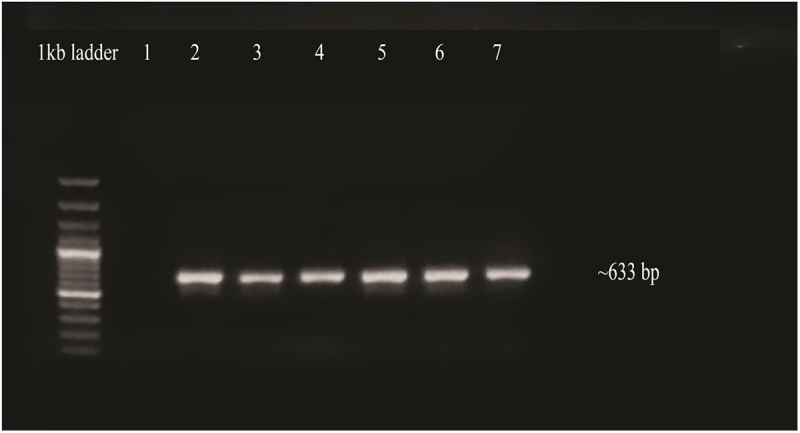
Confirmation of transgenic plants by PCR. Lane 1, negative control, lanes 2 to 5: amplified products of OsMYB48 gene, lane 7: positive control.

To validate transgene insertion, PCR amplification was performed by means of isolated DNA from transgenic plants as a template. The OsMYBF and OsMYBR primers produced high-quality PCR products with a size of approximately 633 bp. The amplified products were then seen and documented on a 1% agarose gel (Figure 11).

## 4. Discussion

MYB TF is a diverse and significant family of transcription factors that govern numerous defense responses to abiotic and biotic stressors, as well as hormone signaling.^24^ The role of MYB TF in the regulation of cellular and metabolic processes in plants is studied at large and found to be promising.^25^ Rice is a major cereal crop all over the world. Understanding the molecular mechanisms of plant MYB TF, regulation of MYB TF during environmental stresses, factors associated with enhanced resistance to abiotic stresses, identification of novel genes, and exploration of existing genetic resources are all important steps in ensuring food security in adverse environmental conditions.^26–29^

In the present study, OsMYB TF from the rice was analyzed. The MYB TF is a highly conserved class of TF, thus its functions are also highly conserved among plants, including both monocots and dicots. In order to evaluate the evolution of OsMYB48 protein in rice and other plant species, a phylogenetic study was performed, and proteins were widely grouped into four groups. A relatively large evolutionary distance was observed between dicots and monocots, but a close relationship between genus *Oryza, Aegliops*, and *Brachypodium* is in line with botanical classification. An interesting phenomenon was observed in sub-class D1, in which a dicot PvMYB was classified with three monocot MYB proteins (Fig. 3). These results are in alignment with the previous study.^30^ This further supports the results obtained in the present study and suggests a further expansion of R2-R3 MYB genes in monocots and dicots. It was also supported by the results obtained after MSA, in which R2-R3 MYB domains were found to be highly conserved among all MYB TFs.

The conserved protein domains and motif analysis results predicted the fundamental role in the functionality of OsMYB48 TF. Gene structure analysis, however, revealed variations among species, which may predict different downstream regulatory genes that take part in multiple signaling pathways. However, species with an approximately similar number of introns grouped together in a phylogenetic tree, supporting the results of this study. It was found that *Brachypodium* has zero introns in its gene structure, but japonica rice showed one intron in its gene structure (Figure 3). The function of OsMYB48 can be determined by the location and characteristics of the cis-elements located in the gene promoter sequence. OsMYB48 is found to be mainly involved in light responses and the regulation of abiotic stress, as shown in Figure 3. To date, there are only a few studies on the regulatory analysis of OsMYB48 TF. Light induces massive reprogramming of the plant transcriptomes and modulates signaling pathways involved in the increase or decrease of gene expression.

Previous and present studies have shown great similarities in amino acid sequence and gene structure between OsMYB48 and its relative closest homologue, OsMYB59.^31^ OsMYB48’s promoter analysis revealed the cis-regulatory elements that are sensitive toward abscisic acid, gibberellin, and methyl jasmonate. It suggests their role in hormonal signaling. The fact that OsMYB48 and other MYB proteins’ subcellular location was predicted to be the nucleus of the cell, suggests their significant role in biological processes. *Agrobacterium*-mediated transformation of gene insertion in a plant is the most reliable and stable process. It was followed for OsMYB48’s transformation in a tobacco plant, under controlled conditions. Hormonal and antibiotic concentrations were increased or decreased to select the transformed plant and improve the transgenic plant’s growth under the light and dark cycle of growth. In a nutshell, OsMYB48 showed a promising role in major cellular processes and defense mechanisms, and its transformation in a model plant could pave the way for its expression analysis in other plants, under various environmental conditions.

Translating the findings from MYB transcription factor research into practical agricultural applications faces several challenges, including the complexity of plant stress responses, potential off-target effects of genome editing, and the regulatory hurdles associated with transgenic crops. Future research should prioritize the development of precise genome editing techniques to minimize off-target effects, comprehensive environmental and ecological impact assessments to address regulatory concerns, and the exploration of non-transgenic genome editing to circumvent the challenges associated with transgenic approaches. Additionally, interdisciplinary collaborations integrating plant physiology, molecular biology, and agronomy are essential to ensure the successful application of these findings in enhancing crop resilience to abiotic stresses.

## Data Availability

All the raw data of this research can be obtained from the corresponding authors upon reasonable request.
